# Structured MOAKS-based MRI features for predicting medial joint space narrowing progression: a comparative osteoarthritis initiative study

**DOI:** 10.3389/fphys.2026.1908547

**Published:** 2026-08-14

**Authors:** Minggui Bao, Ahmad Alkhatatbeh, Jiankun Xu, Hongjiang Chen, Zhonglian Huang, Xiaohui Lu, Tariq Alkhatatbeh, Jun Hu

**Affiliations:** 1 Department of Orthopedics Surgery, The First Affiliated Hospital of Shantou University Medical College, Shantou, China; 2 Department of Orthopaedics & Traumatology, Faculty of Medicine, The Chinese University of Hong Kong, Hong Kong, Hong Kong SAR, China; 3 Department of Sports Orthopaedics, TUM University Hospital, Technical University of Munich, Munich, China; 4 Department of Joint Surgery, Center for Orthopedic Surgery, The Third Affiliated Hospital of Southern Medical University, Guangzhou, China

**Keywords:** joint space narrowing, knee osteoarthritis, MOAKS, MRI, osteoarthritis initiative (OAI)

## Abstract

**Background:**

Knee osteoarthritis (OA) is a major cause of pain and disability, yet baseline radiographic severity grading provides only modest prediction of structural progression. We evaluated whether structured MRI-derived MOAKS features improve prediction of full-grade medial joint space narrowing (JSN) progression, and whether deep multimodal fusion adds value beyond structured feature models.

**Methods:**

We performed a patient-independent internal-validation study using the Osteoarthritis Initiative. Full-grade medial joint-space-narrowing (JSN) progression was defined as an increase of at least one grade between baseline and fixed 48-month central radiographic readings from the same longitudinal reading project. The final analytic cohort included 2,899 knees from 2,392 participants, partitioned at the participant level into training, validation, and held-out test sets of 2,050, 421, and 428 knees. We compared a radiographic baseline model (RBM), raw-MOAKS model (RMO), structured MRI/radiographic model (SMR), and radiograph-plus-MOAKS ResNet50 fusion model (XMF). AUPRC was primary; uncertainty was estimated with 2,000 patient-cluster bootstrap replicates.

**Results:**

Progression prevalence was 10.0%. On the held-out test set (40 progressor knees), AUPRC/AUROC were 0.220 (95% CI 0.142–0.331)/0.748 (0.663–0.827) for RBM, 0.412 (0.260–0.576)/0.805 (0.726–0.878) for RMO, 0.345 (0.219–0.507)/0.801 (0.725–0.871) for SMR, and 0.301 (0.191–0.464)/0.768 (0.679–0.852) for XMF. Compared with RBM, AUPRC increased by 0.192 (0.087–0.313) for RMO and 0.125 (0.042–0.233) for SMR; the corresponding AUROC differences were 0.057 (−0.012 to 0.123) and 0.053 (0.004–0.105). XMF did not improve on SMR in AUPRC or AUROC (differences −0.044 [−0.135 to 0.060] and −0.033 [−0.092 to 0.018], respectively). A stronger clinical-radiographic comparator achieved AUPRC/AUROC 0.251/0.759, increasing to 0.355/0.813 after adding MOAKS.

**Conclusion:**

Baseline MOAKS features provided prognostic information beyond radiographic or clinical-radiographic severity for fixed 48-month medial JSN progression. In this selected MOAKS-scored cohort, adding a baseline-radiograph ResNet50 encoder did not improve discrimination over the structured models. The finite number of test events, selected MOAKS availability, calibration requirements, and lack of external validation preclude claims of clinical readiness.

## Introduction

Knee osteoarthritis (OA) is a common musculoskeletal condition worldwide and a leading cause of chronic pain and functional disability. Globally, an estimated 595 million people, approximately 7.6% of the global population, were living with OA in 2020, a 132% increase over three decades, with knee OA projected to grow a further 74.9% by 2050 as a consequence of population ageing and rising obesity rates (Steinmetz et al., 2023). Structural disease progression, operationally defined as worsening of radiographic joint space narrowing (JSN), signals the transition from early symptomatic disease to end-stage arthritis requiring joint replacement, and its accurate prediction is a prerequisite for designing disease-modifying trials, targeting preventive interventions, and stratifying patients toward unicompartmental or total knee arthroplasty (Steinmetz et al., 2023; Mohammadi et al., 2026). A 2026 systematic review of 99 studies confirmed recurrent prognostic signals for bone marrow lesions (BMLs), meniscal extrusion, synovitis, and cartilage T2 abnormalities, with odds ratios up to 12.5 for bone-shape patterns and 21.3 for cartilage T2 abnormalities, underscoring the multiparametric nature of the progression phenotype (Mohammadi et al., 2026).

Standard radiographic grading, including the Kellgren–Lawrence (KL) grading system and compartment-specific JSN scoring, captures only the downstream bony consequences of articular cartilage loss and is insufficiently sensitive to subclinical structural changes in early and mid-stage OA (Hunter et al., 2011; Collins et al., 2025). Magnetic resonance imaging (MRI) provides direct visualization of cartilage, subchondral bone, menisci, synovium, and osteophytes, enabling richer characterization of joint tissue burden. The MRI Osteoarthritis Knee Score (MOAKS), a standardized semiquantitative instrument covering multiple structural domains, including cartilage damage, BMLs, meniscal morphology, osteophytes, and synovitis/effusion, has shown substantial to near-perfect reliability across most features and has been developed specifically to support OA progression studies (Hunter et al., 2011). In a MOAKS-based cohort enrichment study, MOAKS-defined cartilage damage and BML presence predicted subsequent medial femorotibial cartilage thickness loss with odds ratios of 2.77 and 2.69, respectively, supporting their use as pre-specified predictors rather than exploratory features (Wirth et al., 2023). External validation in the Foundation for the National Institutes of Health (FNIH) Osteoarthritis (OA) Biomarkers Consortium PROGRESS OA study further showed that MRI imaging biomarkers achieved cross-validated AUCs of 0.69 for radiographic and 0.77 for symptomatic progression, while structural phenotype analyses in OAI participants demonstrated associations with both short-term progression and later total knee replacement (Liu et al., 2024; Collins et al., 2025).

Despite a growing body of machine learning literature addressing OA progression, few studies have directly compared structured MRI-derived features with a baseline-radiograph-plus-structured-feature fusion model under the same partitions and endpoint. Machine-learning models integrating multiple MRI pathologies and clinical phenotypes have reported OAI test AUCs of up to 0.837 for joint replacement prediction (D’Assignies et al., 2025). An MRI radiomics framework reported AUC 0.738 for progression stratification (Fu et al., 2025). A 2025 systematic review and meta-analysis pooled MRI-based progression c-indices at 0.798 for MRI-only and 0.806 for MRI plus clinical models, compared with 0.712 for X-ray-based models (Liu et al., 2025). An MRI-based convolutional model using coronal MRI and clinical variables reported AUROC of 0.65 for JSN progression prediction (Schiratti et al., 2021), and a multimodal transformer study still reported moderate discrimination for radiographic progression across longer horizons (Panfilov et al., 2025). AI narrative reviews continue to caution that deep learning models are not uniformly superior to structured feature approaches (Jo and Sebro, 2026). At the technical level, recent segmentation work emphasizes that cartilage and meniscus annotation is still data-intensive and sensitive to domain shift (Ferreira et al., 2025; Khan et al., 2026). Broader AI reviews describe the field as moving from radiomics to end-to-end frameworks, but with task-dependent performance (Ming et al., 2026). Segmentation-error experiments show that downstream OA classifiers can still be perturbed by mask imperfections (Fox et al., 2026). Structural worsening and pain are related but not interchangeable, as shown by the bidirectional OAI knee-pair analysis (Liu et al., 2026).

Quantitative MRI studies continue to mirror these signals, with multiple structural measures achieving AUCs of 0.690 to 0.804 for radiographic progression and performing similarly to semiquantitative scoring (Smith et al., 2025). A supplemented MOAKS approach has also been proposed to capture deep partial-thickness cartilage loss more granularly without abandoning structured interpretation (Altahawi et al., 2025). In OAI cohorts, baseline loss of knee extension has been associated with longitudinal MRI progression patterns, whereas knee stiffness has been associated with MRI-detected meniscus lesions, synovitis, and effusion (Chan Chun Kong et al., 2025; Kong et al., 2025). MRI-defined tibiofemoral OA rarely occurs without cartilage damage, and MOAKS abnormalities can already be associated with symptoms in younger adults (Chang et al., 2026; Kemppainen et al., 2026). Emerging MRI biomarkers such as infrapatellar fat pad changes and thigh muscle radiomics further broaden the landscape, but they also increase feature dimensionality and modeling complexity (Dang et al., 2026; Wang et al., 2026).

The present study addressed this specific comparison. Using the OAI public cohort, we developed and compared four pre-specified models spanning the complexity spectrum from radiographic baseline logistic regression through structured MOAKS elastic-net models to a ResNet-50-based XMF. All models were evaluated by patient-independent train/validation/test splitting and by formal paired bootstrap comparisons on a shared held-out test set. We evaluated whether baseline MOAKS features improved prediction beyond conventional radiographic severity and whether adding a baseline-radiograph ResNet50 encoder to MOAKS and radiographic variables improved discrimination over structured models. End-to-end learning from the source MRI images was not evaluated.

## Methods

### Study design and data source

We performed a retrospective, knee-level internal-validation study using the Osteoarthritis Initiative (OAI) public dataset (Lester, 2016). Baseline radiographs and patient-side mapping were obtained from the Chen-curated OAI image subset available through Mendeley Data (Chen, 2018), which contained 4,130 participants and 8,260 baseline knee observations. Official OAI public releases provided central semiquantitative radiographic readings, demographic variables, knee-specific symptoms, and baseline MRI Osteoarthritis Knee Score (MOAKS) assessments. Records were linked by participant identifier and knee side.

**Figure 1:**
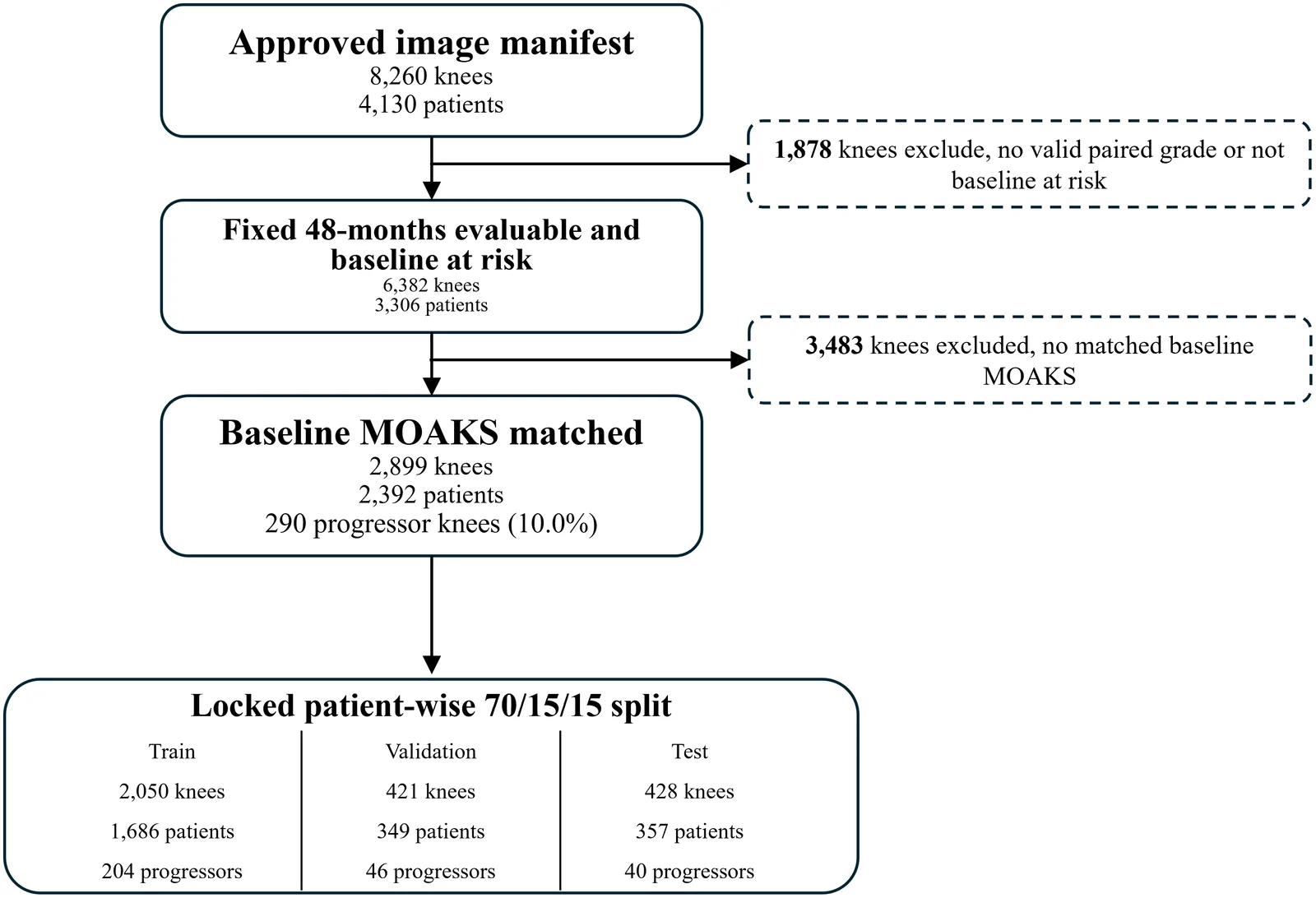
Cohort flow for the fixed 48-month analysis. The approved baseline-image cohort contained 8,260 knees. Paired baseline and 48-month central readings and baseline at-risk criteria yielded 6,382 knees from 3,306 participants; baseline MOAKS matching yielded 2,899 knees from 2,392 participants, including 290 progressor knees. The final participant-level partitions contained 2,050 training, 421 validation, and 428 test knees.

Baseline and fixed 48-month medial JSN grades were obtained from paired central semiquantitative radiographic assessments within the same longitudinal reading project. Knees required numeric medial JSN grades at both visits, baseline medial and lateral JSN grades below 3, and a valid baseline Kellgren–Lawrence grade. These criteria produced 6,382 fixed-48-month-evaluable at-risk knees from 3,306 participants. Matching by participant identifier and knee side to baseline MOAKS assessments yielded the final analytic cohort of 2,899 knees from 2,392 participants; 3,483 otherwise eligible knees lacked a matched baseline MOAKS assessment. The cohort flow is summarized in **Figure 1**.

Baseline MOAKS assessments were obtained from OAI reading projects 22, 30, 46, 47, 63A, 63B, 63D, 63E, 63F, and 65. For duplicate participant-side records, the record with the most complete MOAKS data was retained, followed by reading-project order if completeness was equal. Because several projects were enriched or case-control substudies, the MOAKS-matched cohort should not be considered population-representative.

To assess potential selection associated with MOAKS availability, MOAKS-matched and MOAKS-unavailable at-risk knees were compared on age, sex, body mass index, knee-specific WOMAC pain, knee side, baseline Kellgren–Lawrence and medial/lateral JSN grades, and 48-month progression. Standardized mean differences were used to describe between-group differences. The results are provided in **Supplementary Table 2**.

### Outcome definition

The primary endpoint was fixed 48-month full-grade medial JSN progression. For each knee, the baseline central medial JSN grade was subtracted from the 48-month central medial JSN grade within the same longitudinal reading project. An increase of at least 1.0 grade was classified as progression. Smaller positive decimal changes represented within-grade worsening and were classified as non-events in the primary analysis. Knees without a numeric medial JSN grade at either visit were excluded. In a sensitivity analysis, progression was broadened to any positive change, including within-grade worsening, and the structured models were refitted without changing the predictors or participant assignments. The observed grade transitions and distribution of baseline-to-48-month medial JSN change are provided in **Supplementary Table 3**.

### Predictors and model sets

**Figure 2:**
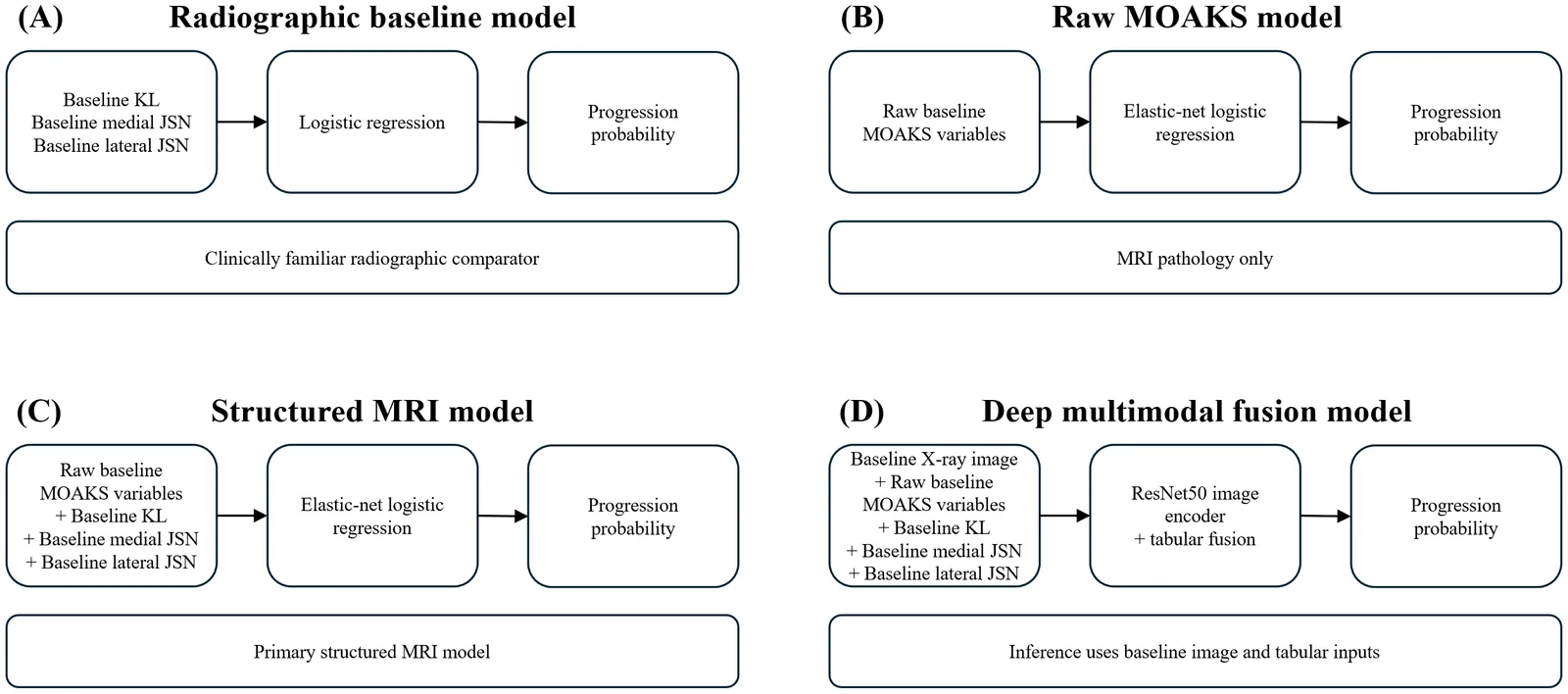
Manuscript model set. **(A)** Radiographic baseline model (RBM) using baseline KL, medial JSN, and lateral JSN. **(B)** Raw MOAKS model (RMO). **(C)** Structured MRI model (SMR) combining raw baseline MOAKS variables with baseline radiographic scores. **(D)** X-ray plus MOAKS fusion model (XMF) using a ResNet-50 image encoder with MOAKS/radiographic fusion.

We compared four pre-specified models spanning increasing levels of imaging complexity. The radiographic baseline model (RBM) used baseline KL grade, baseline medial JSN grade, and baseline lateral JSN grade. The raw MOAKS model (RMO) used the full set of baseline semiquantitative MOAKS variables only, including cartilage, bone marrow lesion, meniscal, osteophyte, and synovitis/effusion features (115 in total). The structured MRI model (SMR) combined the raw MOAKS variables with baseline KL grade, baseline medial JSN grade, and baseline lateral JSN grade. The X-ray plus MOAKS fusion model (XMF) combined the same structured predictors as SMR with baseline knee radiographs processed through a ResNet-50 image encoder. The model set is illustrated in **Figure 2** and summarized in **Table 1B**. For descriptive presentation in **Table 2**, domain-level MOAKS values for cartilage morphology, bone marrow lesions, meniscal findings, osteophytes, and synovitis/effusion were calculated by summing the corresponding item scores. These descriptive domain sums were not used as model predictors; the models used the individual MOAKS items. The complete list of the 115 individual MOAKS predictors is provided in **Supplementary Table 4**.

**Table 1:** Patient-wise split counts and model definitions.

Part A. Shows the locked 70/15/15 patient-wise train/validation/test split.							
Split	Participants	Knees	Progressor knees	Non-progressor knees	Progression prevalence	Participants with >=1 progressor knee	Participants without a progressor knee
Training	1,686	2,050	204	1,846	10.0%	190	1,496
Validation	349	421	46	375	10.9%	41	308
Test	357	428	40	388	9.3%	40	317
Overall	2,392	2,899	290	2,609	10.0%	271	2,121

Part B. Prespecified primary model definitions.							
Model	Model role		Model type		Input features		Selection criterion
RBM	Radiographic baseline comparator		Logistic regression		Baseline KL, medial JSN, and lateral JSN grades		Validation AUPRC
RMO	MOAKS-only structured model		Elastic-net logistic regression		Individual baseline MOAKS variables		Validation AUPRC
SMR	MRI/radiographic structured model		Elastic-net logistic regression		RMO variables plus baseline KL, medial JSN, and lateral JSN grades		Validation AUPRC
XMF	Baseline-radiograph plus structured-feature fusion		ImageNet-pretrained ResNet50 with a tabular branch		Baseline radiograph plus SMR structured features; no raw MRI input		Validation AUPRC

No patient overlap was present across splits. RBM, radiographic baseline model; RMO, raw MOAKS model; SMR, structured MRI model; XMF, X-ray plus MOAKS fusion model; LR, logistic regression.

**Table 2:** Baseline characteristics of the final MOAKS-matched at-risk cohort by progression status.

Characteristic	Overall	Progressor	Non-progressor	SMD
Knees, n	2,899	290	2,609	Not calculated
Participants, n	2,392	271	2,177	Not calculated
Age, years, mean (SD)	60.276 (8.980)	61.224 (8.190)	60.170 (9.059)	0.122
Sex, n (%)	Male: 1,178 (40.6%) Female: 1,721 (59.4%)	Male: 121 (41.7%) Female: 169 (58.3%)	Male: 1,057 (40.5%) Female: 1,552 (59.5%)	0.025
BMI, kg/m^2, mean (SD)	28.192 (4.678)	30.227 (4.838)	27.966 (4.605)	0.479
Knee side, n (%)	Left: 1,052 (36.3%) Right: 1,847 (63.7%)	Left: 127 (43.8%) Right: 163 (56.2%)	Left: 925 (35.5%) Right: 1,684 (64.5%)	0.171
Baseline KL grade, n (%)	0: 1,443 (49.8%) 1: 674 (23.2%) 2: 479 (16.5%) 3: 303 (10.5%)	0: 61 (21.0%) 1: 76 (26.2%) 2: 94 (32.4%) 3: 59 (20.3%)	0: 1,382 (53.0%) 1: 598 (22.9%) 2: 385 (14.8%) 3: 244 (9.4%)	Not calculated
Baseline medial JSN grade, n (%)	0: 2,093 (72.2%) 1: 556 (19.2%) 2: 250 (8.6%)	0: 135 (46.6%) 1: 96 (33.1%) 2: 59 (20.3%)	0: 1,958 (75.0%) 1: 460 (17.6%) 2: 191 (7.3%)	Not calculated
Baseline lateral JSN grade, n (%)	0: 2,726 (94.0%) 1: 117 (4.0%) 2: 56 (1.9%)	0: 282 (97.2%) 1: 8 (2.8%) 2: 0 (0.0%)	0: 2,444 (93.7%) 1: 109 (4.2%) 2: 56 (2.1%)	Not calculated
MOAKS cartilage-morphology severity sum, median [IQR]	4.100 [2.000, 8.000]	7.450 [4.125, 11.375]	4.000 [1.100, 7.200]	0.649
MOAKS subregional BML severity sum, median [IQR]	5.000 [0.000, 10.000]	10.500 [4.000, 19.000]	4.000 [0.000, 10.000]	0.691
MOAKS meniscal-findings severity sum, median [IQR]	2.000 [0.000, 6.000]	5.000 [2.000, 9.000]	2.000 [0.000, 6.000]	0.476
MOAKS osteophyte severity sum, median [IQR]	1.000 [0.000, 4.000]	6.000 [2.000, 11.250]	1.000 [0.000, 3.000]	0.866
MOAKS synovitis/effusion severity sum, median [IQR]	1.000 [0.000, 1.000]	1.000 [1.000, 2.000]	1.000 [0.000, 1.000]	0.675

Values are mean (SD), median [IQR], or n (%). SMDs were not calculated for multilevel KL or JSN distributions. Outcome-group participant counts are not mutually exclusive because bilateral knees may have different outcomes. BMI was missing for one knee. Laterality imbalance likely reflects reading-project sampling and should not be interpreted causally. BMI, body mass index; BML, bone marrow lesion; IQR, interquartile range; JSN, joint-space narrowing; KL, Kellgren–Lawrence; MOAKS, MRI Osteoarthritis Knee Score; SD, standard deviation; SMD, standardized mean difference.

### Split strategy and leakage prevention

The final analytic cohort was partitioned at the participant level into training (70%), validation (15%), and test (15%) sets, with the partition fixed before model fitting. Both knees from the same participant were assigned to the same partition. Models were fitted using the training set; the validation set was used for hyperparameter and model selection, recalibration fitting, and operating-threshold selection; and the test set was used for performance evaluation. The training, validation, and test sets contained 2,050, 421, and 428 knees from 1,686, 349, and 357 participants, respectively, including 204, 46, and 40 progressor knees. No participant appeared in more than one partition.

### Feature preprocessing and tabular model development

RBM, RMO, and SMR were trained as supervised binary classifiers using the training split and tuned on the validation split. Continuous predictors were median-imputed and standardized using parameters estimated from the training data only. Ordinal grades were retained in their original coded order and treated as numeric predictors before training-set median imputation and standardization. RBM used standard logistic regression. RMO and SMR used elastic-net logistic regression to combine L1 and L2 regularization, allowing shrinkage and sparse feature selection across the larger MOAKS feature set.

For the elastic-net models, the regularization strength *C* was searched over {0.01, 0.1, 0.3, 1.0, 3.0, 10.0}, and the *l1 ratio* was searched over {0.1, 0.5, 0.9}. Class imbalance was addressed using inverse-frequency class weighting in the tabular classifiers. Hyperparameter selection for all tabular models was based on validation AUPRC. The final fitted model for each approach used the hyperparameter combination with the best validation AUPRC. A probability threshold of 0.50 was retained as a descriptive analysis; validation-selected operating thresholds were selected using validation predictions and applied without modification to the test set.

As an ordinal-coding sensitivity analysis, MOAKS and radiographic grades were represented by one-hot indicator variables after most-frequent-category imputation and fitted using class-weighted ridge logistic regression. Regularization strength was selected by validation AUPRC; the numeric-ordinal models remained the primary analysis.

### Radiograph–structured feature fusion model development

XMF used an ImageNet-pretrained ResNet-50 image encoder with a parallel tabular branch for the structured predictors. Baseline radiographs were resized to 224 × 224 pixels. During training, the image branch used random resized cropping, and light color jittering. Validation and test images were processed using deterministic resize-normalization. The image embedding was concatenated with the tabular embedding and passed to a binary classifier head.

To address class imbalance, XMF was trained using class-weighted binary cross-entropy, giving progressor knees proportionally greater contribution to the loss. Optimization used AdamW, with a lower learning rate for the image backbone than for the fusion head, weight decay of 1e-4, and batch size of 32. A ReduceLROnPlateau scheduler was applied, and early stopping was used after 5 epochs without validation AUPRC improvement, with a maximum of 20 training epochs. The checkpoint with the best validation AUPRC was retained for test evaluation. Models were implemented in Python using PyTorch and torchvision.

### Secondary comparators and sensitivity analyses

An enhanced clinical-radiographic comparator included age, sex, body mass index, knee-specific baseline WOMAC pain, Kellgren–Lawrence grade, and medial and lateral JSN grades. Continuous variables were median-imputed and standardized, and sex was one-hot encoded after most-frequent-category imputation. This comparator used class-weighted ridge logistic regression. A corresponding elastic-net model added the same individual MOAKS predictors used in RMO. Hyperparameters were selected by validation AUPRC.

To examine the roles of model architecture and input modality, two neural-network ablations were evaluated. A tabular multilayer perceptron used the same MOAKS and radiographic predictors as SMR, with hidden layers of 128 and 64 units. An X-ray-only model used the same ResNet50 image branch and preprocessing as XMF but omitted the tabular predictors. Both used class-weighted binary cross-entropy, AdamW optimization, validation-AUPRC model selection, and early stopping. These analyses were secondary and architecture-specific.

For endpoint sensitivity, progression was broadened to any positive baseline-to-48-month medial JSN change, including within-grade worsening. RBM, RMO, and SMR were refitted using the unchanged predictors and participant partitions.

### Performance evaluation

The primary discrimination metric was area under the precision–recall curve (AUPRC), calculated as average precision and selected *a priori* because progression was uncommon. Area under the receiver-operating-characteristic curve (AUROC) was reported as a complementary threshold-independent metric. Brier score summarized probabilistic accuracy. ROC and precision–recall curves were generated on the fixed test partition. Calibration was assessed using quantile-binned calibration curves and calibration intercept and slope, estimated by logistic regression of outcome on the logit of predicted probability. For RMO and SMR, logistic recalibration was fitted using validation predictions and then applied without refitting to test predictions.

Thresholds corresponding to the validation Youden index, approximately 90% sensitivity, and approximately 90% specificity were selected from validation predictions and locked before test evaluation. Sensitivity, specificity, positive predictive value, and negative predictive value were then calculated in the test set. Predictive values were interpreted as specific to the prevalence of the selected MOAKS-scored cohort.

### Statistical comparison and uncertainty estimation

Confidence intervals for individual model estimates and pairwise model differences were calculated using 2,000 patient-level cluster bootstrap replicates. Participants, rather than individual knees, were sampled with replacement, and all eligible knees from each sampled participant were retained. For paired comparisons, the same sampled participant clusters were applied to both models. AUPRC, AUROC, and Brier score were recomputed in each replicate, and 95% percentile confidence intervals were reported. Differences whose 95% confidence intervals excluded zero were considered statistically supported. For Brier score, negative differences indicated improvement because lower values are better. Primary comparisons were prespecified; secondary comparisons were exploratory and were not adjusted for multiplicity. Key comparisons are summarized in **Table 3**.

**Table 3:** Paired patient-cluster bootstrap comparisons on the held-out fixed 48-month test partition.

Contrast	AUPRC difference (95% CI)	AUROC difference (95% CI)	Brier-score difference (95% CI)
RMO - RBM	+0.192 (+0.087 to +0.313)	+0.057 (-0.012 to +0.123)	-0.022 (-0.033 to -0.011)
SMR - RBM	+0.125 (+0.042 to +0.233)	+0.053 (+0.004 to +0.105)	-0.015 (-0.023 to -0.006)
SMR - RMO	-0.067 (-0.117 to -0.020)	-0.004 (-0.031 to +0.027)	+0.008 (+0.004 to +0.012)
XMF - SMR	-0.044 (-0.135 to +0.060)	-0.033 (-0.092 to +0.018)	-0.011 (-0.021 to -0.0004)

Differences are calculated as the first model minus the second model. Positive AUPRC or AUROC differences favor the first model; negative Brier-score differences favor the first model. Confidence intervals are 95% percentile intervals from 2,000 paired patient-level cluster bootstrap replicates. RMO versus RBM, SMR versus RBM, and XMF versus SMR were prespecified primary contrasts. SMR versus RMO was exploratory. Secondary comparisons were not adjusted for multiplicity. AUPRC, area under the precision–recall curve; AUROC, area under the receiver-operating-characteristic curve; CI, confidence interval; RBM, radiographic baseline model; RMO, raw MOAKS model; SMR, structured MRI/radiographic model; XMF, X-ray plus MOAKS fusion model.

### Radiograph-branch explainability

To provide qualitative localization for the radiograph branch of XMF, Gradient-weighted Class Activation Mapping (Grad-CAM) was applied to the final convolutional block of the ResNet50 encoder. Four illustrative test cases were selected deterministically: the highest-probability true-positive and false-positive cases and the lowest-probability true-negative and false-negative cases, with classification defined using the XMF threshold selected by the Youden criterion in the validation set. Grad-CAM reflects only the radiograph branch and does not attribute the tabular MOAKS or radiographic-grade inputs. The maps were interpreted as qualitative localization aids and not as pixel-level segmentations or causal explanations of model reasoning.

### Ethics and reporting

This study is a secondary analysis of the de-identified Osteoarthritis Initiative public dataset, which was conducted under institutional oversight with participants providing written informed consent at the time of original enrolment; no additional ethical approval was required for this secondary analysis. The study is reported in a manner consistent with standard prediction-model reporting practices, with explicit separation of training, validation, and test evaluation; model selection, recalibration fitting, and threshold selection were performed using training or validation data before performance was reported on the fixed test partition.

## Results

### Cohort characteristics

The final analytic cohort contained 2,899 knees from 2,392 participants, including 290 progressor knees (10.0%) and 2,609 non-progressor knees (**Table 2**). Progressor knees had higher BMI than non-progressor knees (30.227 ± 4.838 vs 27.966 ± 4.605 kg/m²; SMD 0.479) and were more often left-sided (43.8% vs 35.5%; SMD 0.171). The laterality difference has no clear biological explanation and is likely related to reading-project sampling. Progressor knees also had greater baseline radiographic and MOAKS burden: KL grade 3 was present in 20.3% versus 9.4%, medial JSN grade 0 in 46.6% versus 75.0%, the cartilage-morphology severity sum was 7.450 [4.125–11.375] versus 4.000 [1.100–7.200], and the subregional BML severity sum was 10.500 [4.000–19.000] versus 4.000 [0.000–10.000].

Among 6,382 fixed-48-month-evaluable at-risk knees, 2,899 had matched baseline MOAKS assessments and 3,483 did not (**Supplementary Table 2**). Progression prevalence was 10.0% in the MOAKS-matched group and 5.6% in the MOAKS-unavailable group. Material differences included knee side (maximum absolute SMD 0.518), KL grade 2 (SMD −0.460), KL grade 0 (SMD 0.342), and progression status (SMD 0.166), whereas differences in age, sex, BMI, and WOMAC pain were small. These findings indicate selected MOAKS availability and limit generalizability of absolute performance estimates.

The fixed participant-level partitions contained 2,050 training knees with 204 progressors (10.0%), 421 validation knees with 46 progressors (10.9%), and 428 test knees with 40 progressors (9.3%) (**Table 1A**). No participant overlap was present across partitions. Model definitions are shown in **Table 1B**.

### Model discrimination

**Figure 3:**
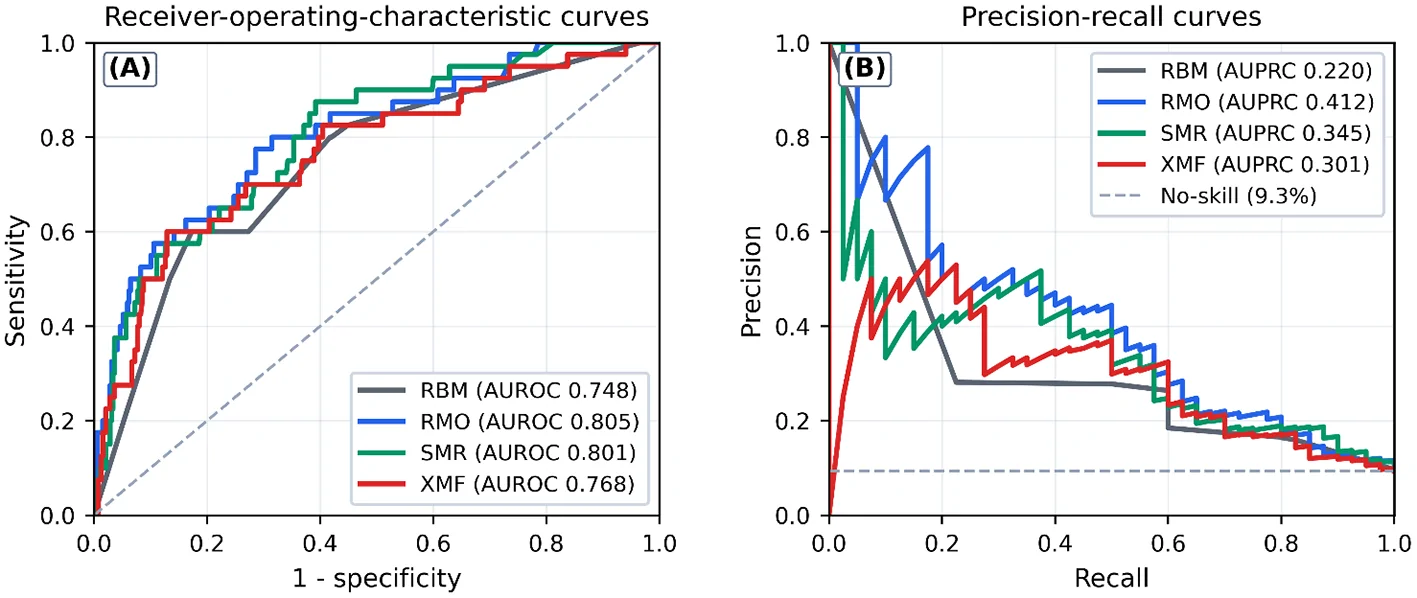
Discrimination on the shared held-out test split. **(A)** ROC curves for RBM, RMO, SMR, and XMF. **(B)** Precision-recall curves for the same models, with the no-skill reference line shown at the test-set progression prevalence (9.3%).

Model discrimination on the fixed test partition is shown in **Table 4** and **Figure 3**. RBM achieved AUPRC 0.220 (95% patient-cluster CI 0.142–0.331) and AUROC 0.748 (0.663–0.827). RMO had the highest AUPRC at 0.412 (0.260–0.576) and AUROC 0.805 (0.726–0.878). SMR achieved AUPRC 0.345 (0.219–0.507) and AUROC 0.801 (0.725–0.871), while XMF achieved AUPRC 0.301 (0.191–0.464) and AUROC 0.768 (0.679–0.852). Thus, RMO and SMR had statistically supported increases in AUPRC over RBM, while XMF showed no evidence of improved AUPRC or AUROC over SMR.

**Table 4:** Discrimination and Brier scores on the held-out fixed 48-month test partition.

Model	Test AUPRC (95% CI)	Test AUROC (95% CI)	Test Brier score (95% CI)
RBM	0.220 (0.142 to 0.331)	0.748 (0.663 to 0.827)	0.210 (0.197 to 0.224)
RMO	0.412 (0.260 to 0.576)	0.805 (0.726 to 0.878)	0.188 (0.174 to 0.201)
SMR	0.345 (0.219 to 0.507)	0.801 (0.725 to 0.871)	0.196 (0.183 to 0.208)
XMF	0.301 (0.191 to 0.464)	0.768 (0.679 to 0.852)	0.185 (0.168 to 0.202)

AUPRC was the prespecified primary discrimination metric. Values in parentheses are 95% percentile confidence intervals from 2,000 patient-level cluster bootstrap replicates. Lower Brier scores indicate better accuracy of the raw model probabilities. AUPRC, area under the precision–recall curve; AUROC, area under the receiver-operating-characteristic curve; CI, confidence interval; RBM, radiographic baseline model; RMO, raw MOAKS model; SMR, structured MRI/radiographic model; XMF, X-ray plus MOAKS fusion model.

### Calibration and threshold-based performance

**Figure 4:**
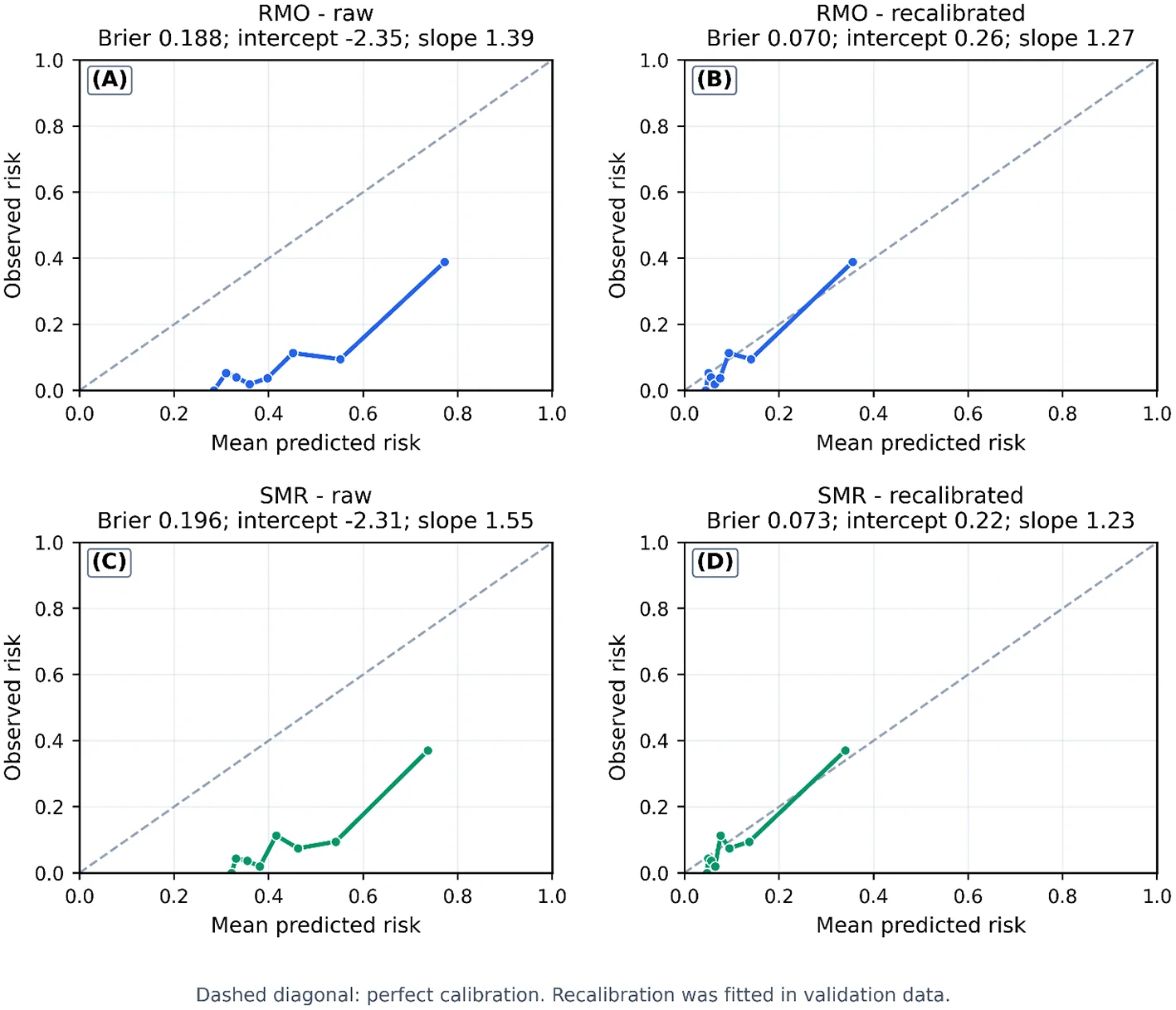
Calibration of the structured MOAKS models on the shared held-out test set. **(A)** RMO raw predictions. **(B)** RMO predictions after logistic recalibration fitted using validation data. **(C)** SMR raw predictions. **(D)** SMR predictions after logistic recalibration fitted using validation data. Points represent quantile-binned observed versus predicted probabilities, and the dashed diagonal indicates perfect calibration.

Raw class-weighted probabilities overestimated absolute risk. In the test set, mean predicted risk was 43.2% for RMO and 44.3% for SMR versus an observed prevalence of 9.3%. Raw calibration intercept/slope were −2.35/1.39 for RMO and −2.31/1.55 for SMR. Logistic recalibration fitted in validation data reduced test Brier scores from 0.188 to 0.070 for RMO and from 0.196 to 0.073 for SMR and brought mean predicted risk to approximately 11% (**Figure 4**). These recalibrated probabilities remain internally validated only.

At validation-selected operating points, the RMO Youden threshold of 0.472 produced sensitivity 0.650, specificity 0.763, PPV 0.220, and NPV 0.955 in the test set. An RMO threshold targeting high specificity (0.606) produced sensitivity 0.550, specificity 0.897, PPV 0.355, and NPV 0.951. An SMR threshold targeting high sensitivity (0.357) produced sensitivity 0.950, specificity 0.351, PPV 0.131, and NPV 0.986. PPV and NPV are prevalence-dependent and apply to this selected MOAKS-scored cohort.

### Paired bootstrap comparisons

Patient-cluster paired bootstrap comparisons are summarized in **Table 3**. Compared with RBM, AUPRC was higher for RMO by 0.192 (95% CI 0.087–0.313) and for SMR by 0.125 (0.042–0.233). RMO had higher AUPRC than SMR by 0.067 (0.020–0.117), whereas their AUROCs did not differ clearly. XMF did not improve on SMR in AUPRC (difference −0.044, −0.135 to 0.060) or AUROC (−0.033, −0.092 to 0.018). Secondary comparisons were exploratory and unadjusted for multiplicity.

### Secondary and sensitivity analyses

The enhanced clinical-radiographic comparator achieved test AUPRC 0.251 and AUROC 0.759. Adding MOAKS increased AUPRC to 0.355 (difference 0.104, 95% CI 0.026–0.215) and AUROC to 0.813 (difference 0.053, 0.002–0.105), supporting incremental prognostic information from MOAKS beyond routinely available clinical and radiographic variables.

The tabular multilayer perceptron achieved AUPRC 0.305 and AUROC 0.753, and the X-ray-only ResNet50 achieved AUPRC 0.209 and AUROC 0.696. The X-ray-plus-MOAKS fusion model achieved AUPRC 0.301 and AUROC 0.768. These ablations are consistent with the fusion result being specific to the tested architecture and data setting; they do not establish a general limitation of deep learning. With categorical encoding, RMO and SMR achieved AUPRC 0.368 and 0.417, respectively. Broadening the endpoint to partial-or-full worsening increased the number of events to 395 (13.6%); after refitting, test AUPRC was 0.347 for RBM, 0.579 for RMO, and 0.530 for SMR. These sensitivity analyses continued to support the prognostic contribution of MOAKS, although the exact RMO/SMR ranking varied with coding. Detailed results for the enhanced comparator, predictor-coding sensitivity, neural-network ablations, and endpoint sensitivity are provided in **Supplementary Table 5**.

### Radiograph-branch interpretability

**Figure 5:**
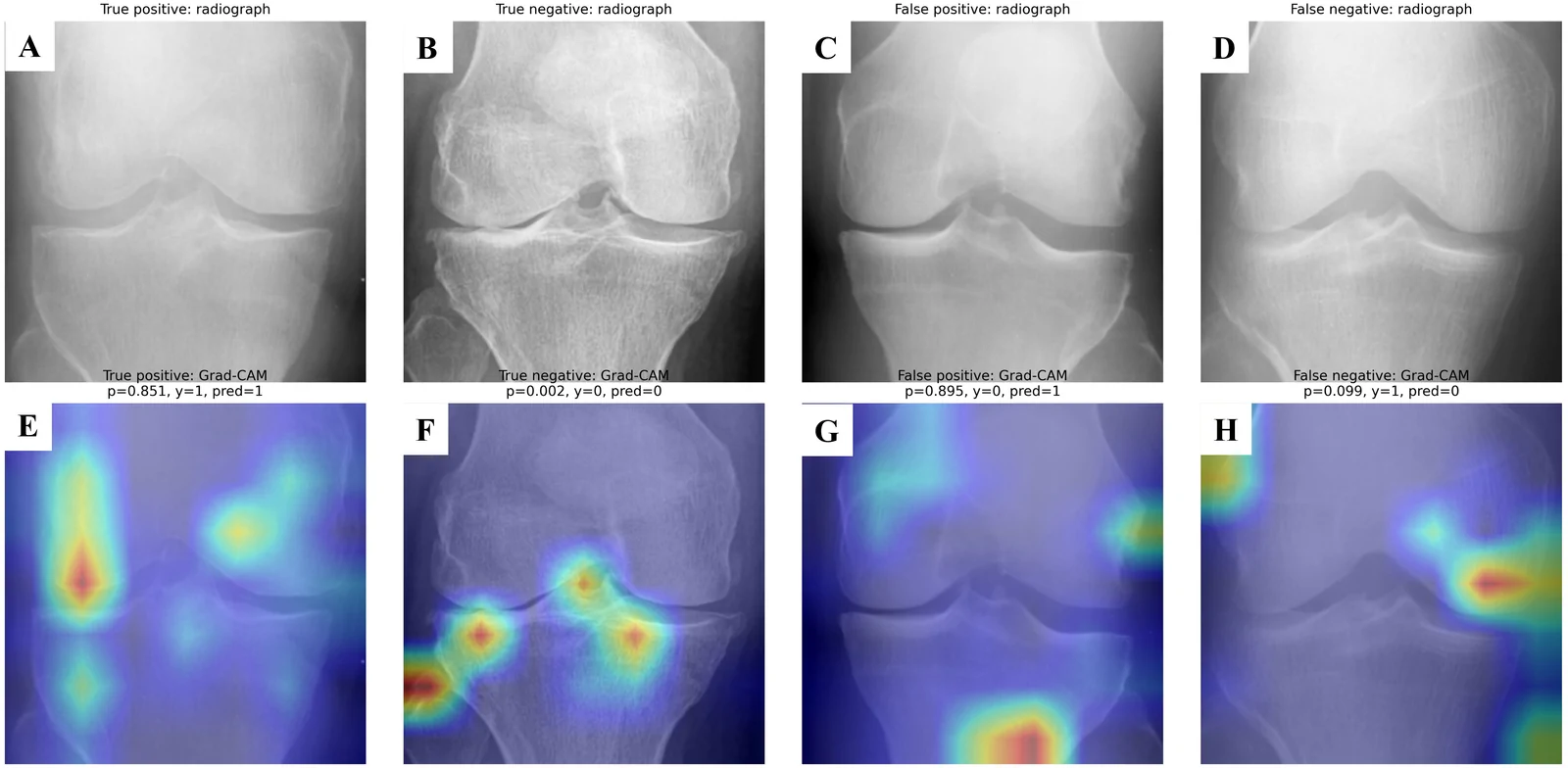
Representative XMF Grad-CAM examples on the shared held-out test split. Panels **(A–D)** show original images and panels **(E–H)** show the corresponding Grad-CAM overlays. **(A, E)** true positive. **(B, F)** true negative. **(C, G)** false positive. **(D, H)** false negative. The highlighted region is a rough joint-space localization aid rather than a segmentation-derived measurement.

Illustrative Grad-CAM maps from XMF are shown in **Figure 5**. The selected true-positive and true-negative maps showed greater activation within or adjacent to the central tibiofemoral region, whereas the false-positive and false-negative maps showed more peripheral activation. Because these were four deterministically selected illustrative cases, the patterns provide only a qualitative sanity check of the radiograph branch; they do not explain the contribution of the tabular inputs or establish stable anatomy-specific model reasoning.

## Discussion

### Main finding

In this patient-independent internal validation, baseline MOAKS features improved prediction of fixed 48-month full-grade medial JSN progression beyond conventional radiographic severity. On the fixed test partition, AUPRC/AUROC were 0.220/0.748 for RBM, 0.412/0.805 for RMO, 0.345/0.801 for SMR, and 0.301/0.768 for XMF. Patient-cluster comparisons supported higher AUPRC for both RMO and SMR than for RBM. The enhanced clinical-radiographic comparator achieved AUPRC/AUROC of 0.251/0.759, increasing to 0.355/0.813 after adding MOAKS.

AUPRC was the primary discrimination metric because progressors comprised only 9.3% of the test set and the precision–recall trade-off directly reflects retrieval of the positive class; AUROC was interpreted as a complementary measure of overall ranking. Their absolute values are on different scales and should not be compared directly. RMO and SMR had nearly identical AUROC but different AUPRC under the primary numeric coding, indicating similar overall rank discrimination but a more favorable precision–recall trade-off for RMO. Categorical coding reversed their numerical AUPRC ranking, however, showing that their relative performance depended on predictor representation. XMF did not improve AUPRC or AUROC over SMR, although the paired confidence intervals for both differences included zero. These findings apply to the selected MOAKS-scored cohort and the specific radiograph-fusion architecture tested; they do not address end-to-end learning from the source MRI images.

### Contextualization against prior work

The discrimination achieved by the MOAKS-based models falls within ranges reported in prior work, although direct numerical comparison is limited by differences in endpoints, cohorts, and validation designs. A 2025 systematic review and meta-analysis pooled MRI-based progression c-indices at 0.798 for MRI-only models and 0.806 for MRI plus clinical models, compared with 0.712 for X-ray-based models (Liu et al., 2025); in the present study, AUROC was 0.805 for RMO and 0.748 for RBM, but these values should not be interpreted as a direct replication of pooled estimates from heterogeneous studies.

External validation of imaging biomarkers in the FNIH PROGRESS OA study reported cross-validated AUCs of 0.69 for radiographic and 0.77 for symptomatic progression (Collins et al., 2025), also consistent with our RBM and MOAKS-based results. In the OAI, MOAKS-defined structural phenotypes characterized by subchondral bone or inflammatory patterns were associated with short-term JSN progression (ORs 1.52–2.15) and with later total knee replacement (HRs 1.71–2.00) (Liu et al., 2024), providing biological grounding for the predictive signal captured by MOAKS domains in our elastic-net model. Quantitative MRI case-control work reported AUCs ranging from 0.690 to 0.804 and found discrimination similar to semiquantitative scoring (Smith et al., 2025). MOAKS-based enrichment work also showed that cartilage damage and BMLs were among the strongest predictors of subsequent cartilage thickness loss (Wirth et al., 2023).

More recent work further supports the clinical centrality of structured MRI findings. The sMOAKS supplement was proposed to capture deep partial-thickness cartilage loss more granularly (Altahawi et al., 2025), and MRI-defined tibiofemoral OA rarely occurs without cartilage damage (Chang et al., 2026). In younger adults, MOAKS abnormalities are already associated with symptoms (Kemppainen et al., 2026). Baseline loss of knee extension has been associated with longitudinal MRI progression patterns, whereas knee stiffness has been associated with MRI-detected meniscus lesions, synovitis, and effusion (Chan Chun Kong et al., 2025; Kong et al., 2025). Structural worsening and pain are related but not interchangeable, as shown by the bidirectional OAI knee-pair analysis (Liu et al., 2026).

Comparison with deep-learning studies requires attention to differences in inputs, endpoints, follow-up horizons, and validation designs. Schiratti and colleagues used a convolutional model with coronal MRI and clinical variables to predict 12-month JSN progression and reported AUROC 0.65; the radiologist benchmark was approximately 0.59 on the same task (Schiratti et al., 2021). A multimodal transformer study reported ROC AUC of 0.70–0.76 across 2–8-year horizons (Panfilov et al., 2025). Studies predicting total knee replacement have reported higher AUCs, but total knee replacement is a different, clinically selected endpoint, so those results should not be ranked directly against JSN-grade progression (D’Assignies et al., 2025; Liu et al., 2025).

The present study provides a direct comparison of structured MOAKS models and a baseline-radiograph ResNet50 fusion model under the same participant partitions and endpoint. The fusion model did not improve discrimination over SMR, but the confidence intervals for XMF-versus-SMR AUPRC and AUROC differences included zero. This architecture-specific result does not address end-to-end learning from the source MRI images.

### Proposed mechanisms for deep fusion underperformance

Several factors may explain why the tested XMF did not improve discrimination over the structured models. First, the training set contained 2,050 knees and 204 progressor knees, which limits estimation of a high-capacity radiograph encoder. Second, MOAKS variables explicitly encode cartilage damage, bone marrow lesions, meniscal abnormalities, osteophytes, and inflammatory features relevant to JSN progression, leaving limited residual information for the radiograph branch. Third, joint optimization of image and tabular branches may favor the more rapidly converging structured branch. The X-ray-only and tabular neural-network ablations support interpreting the result as specific to this radiograph-fusion configuration. These findings should also be considered in the broader context of MRI-based modeling. Knee MRI segmentation and annotation remain data-intensive and can be affected by inter-reader variability and differences between acquisition domains (Ferreira et al., 2025; Khan et al., 2026), while the robustness of classifiers that depend on segmented imaging features requires evaluation under mask errors (Fox et al., 2026). These segmentation-related issues do not directly explain the present XMF result because XMF used baseline radiographs without an explicit segmentation mask. Other MRI approaches using infrapatellar fat pad features and thigh-muscle radiomics may provide complementary prognostic information (Dang et al., 2026; Wang et al., 2026), but they were not evaluated here. End-to-end deep learning from the source MRI images therefore remains an open question.

### Limitations

Several limitations require emphasis. First, baseline MOAKS assessments were available only in selected OAI reading projects, including enriched and case-control substudies. MOAKS-matched and MOAKS-unavailable knees differed in laterality, baseline radiographic severity, and progression prevalence; therefore, the analytic cohort is not population-representative, and selection may influence absolute performance and model comparisons. Second, this was internal validation in a single cohort without external or prospective validation. Third, only 40 progressor knees were present in the fixed test partition, producing wide confidence intervals. Fourth, class-weighted raw probabilities substantially overestimated absolute risk. Validation-fitted recalibration improved internal calibration, but transportability of recalibrated risks and operating thresholds is unknown. Fifth, the primary full-grade endpoint excluded within-grade worsening, although the broader endpoint sensitivity analysis supported the main conclusion. Sixth, Grad-CAM was limited to four deterministically selected illustrative cases from the radiograph branch and lacked segmentation-based localization validation; it therefore cannot explain the tabular contribution or establish causal model reasoning. Finally, the endpoint was radiographic and did not measure symptomatic progression; PPV and NPV are prevalence-dependent and may differ in other settings.

## Conclusion

Baseline MOAKS features provided prognostic information beyond conventional radiographic and clinical-radiographic severity for fixed 48-month medial JSN progression in this selected MOAKS-scored OAI cohort. RMO achieved the highest primary test AUPRC among the four manuscript-facing models. Adding a baseline-radiograph ResNet50 encoder to MOAKS and radiographic variables did not improve discrimination over the structured models in this cohort. This result does not establish that deep learning from MRI is inferior because end-to-end MRI learning was not evaluated. The selected cohort, limited number of test events, internal-only recalibration, and absence of external validation preclude claims of clinical readiness.

## Data Availability

The datasets presented in this study can be found in online repositories. The names of the repository/repositories and accession number(s) can be found below: https://nda.nih.gov/oai.
