# Supplementary material for: Structured MOAKS-based MRI features for predicting medial joint space narrowing progression: a comparative osteoarthritis initiative study
Source: Front Physiol. 2026 Aug 14;17:1908547. doi: 10.3389/fphys.2026.1908547 (PMC13521763; doi:10.3389/fphys.2026.1908547)
Supplement: Supplementary file 1 [file Table1.docx]

**Supplementary Table S1. Baseline MOAKS reading-project composition and contribution to the fixed 48-month analytic cohort.**

| **OAI reading project** | **Released baseline rows** | **Participants in released project** | **Final analytic knees assigned to project** | **Participants contributing final knees** | **Project description** |
| --- | --- | --- | --- | --- | --- |
| 22 | 600 | 600 | 501 | 501 | FNIH OA Biomarkers Consortium release |
| 30 | 600 | 574 | 262 | 254 | Matched case-control study of isolated lateral OA, isolated medial OA, and no radiographic OA |
| 46 | 1,253 | 1,253 | 926 | 926 | Baseline MOAKS release; no separate description was identified in the supplied project-methods document |
| 47 | 860 | 860 | 517 | 517 | Baseline MOAKS release; no separate description was identified in the supplied project-methods document |
| 63A | 110 | 105 | 18 | 18 | Incident radiographic OA cases by 12 months |
| 63B | 61 | 59 | 1 | 1 | Incident radiographic OA cases by 24 months |
| 63C | 63 | 59 | 0 | 0 | Incident radiographic OA cases by 36 months; no knees remained in the final cohort |
| 63D | 37 | 36 | 1 | 1 | Incident radiographic OA cases by 48 months |
| 63E | 308 | 301 | 110 | 109 | Controls without radiographic OA |
| 63F | 81 | 81 | 47 | 47 | Controls with baseline radiographic OA (KL grade 2) |
| 65 | 1,145 | 1,033 | 516 | 491 | POMA incident-OA and knee-replacement nested matched case-control cohorts |

***Note:*** Duplicate participant-side records were resolved by retaining the record with the most complete MOAKS data, followed by reading-project order when completeness was equal. Project-level participant counts are not additive because a participant could contribute knees associated with different projects before participant-side deduplication. Projects 22, 30, 46, 47, 63A, 63B, 63D, 63E, 63F, and 65 contributed knees to the final analytic cohort. KL, Kellgren–Lawrence; MOAKS, MRI Osteoarthritis Knee Score; OA, osteoarthritis; OAI, Osteoarthritis Initiative.

**Supplementary Table S2. Comparison of fixed-48-month-evaluable at-risk knees with and without matched baseline MOAKS assessments.**

| Characteristic | MOAKS matched | MOAKS unavailable | SMD |
| --- | --- | --- | --- |
| Knees, n | 2,899 | 3,483 | Not calculated |
| Participants represented, n | 2,392 | 2,689 | Not calculated |
| Age, years, mean (SD) | 60.28 (8.98) | 61.00 (9.16) | −0.080 |
| Female sex, n (%) | 1,721 (59.4%) | 1,973 (56.6%) | 0.055 |
| BMI, kg/m², mean (SD) | 28.19 (4.68) | 28.53 (4.69) | −0.072 |
| Baseline knee-specific WOMAC pain, mean (SD) | 2.01 (2.97) | 2.10 (3.14) | −0.030 |
| Left knee, n (%) | 1,052 (36.3%) | 2,137 (61.4%) | −0.518 |
| Baseline KL grade 0, n (%) | 1,443 (49.8%) | 1,155 (33.2%) | 0.342 |
| Baseline KL grade 1, n (%) | 674 (23.2%) | 581 (16.7%) | 0.165 |
| Baseline KL grade 2, n (%) | 479 (16.5%) | 1,264 (36.3%) | −0.460 |
| Baseline KL grade 3, n (%) | 303 (10.5%) | 483 (13.9%) | −0.105 |
| Baseline medial JSN grade 0, n (%) | 2,093 (72.2%) | 2,230 (64.0%) | 0.176 |
| Baseline medial JSN grade 1, n (%) | 556 (19.2%) | 873 (25.1%) | −0.142 |
| Baseline medial JSN grade 2, n (%) | 250 (8.6%) | 380 (10.9%) | −0.077 |
| Baseline lateral JSN grade 0, n (%) | 2,726 (94.0%) | 3,220 (92.4%) | 0.063 |
| Baseline lateral JSN grade 1, n (%) | 117 (4.0%) | 151 (4.3%) | −0.015 |
| Baseline lateral JSN grade 2, n (%) | 56 (1.9%) | 112 (3.2%) | −0.081 |
| Full-grade medial JSN progression, n (%) | 290 (10.0%) | 194 (5.6%) | 0.166 |

***Note:*** SMDs are oriented as MOAKS-matched minus MOAKS-unavailable. Participant counts represent participants contributing at least one knee to the respective knee-level group and are not mutually exclusive because one participant could have a MOAKS-matched knee and a contralateral MOAKS-unavailable knee. The largest differences involved laterality, baseline radiographic severity, and progression prevalence. BMI, body mass index; JSN, joint-space narrowing; KL, Kellgren–Lawrence; MOAKS, MRI Osteoarthritis Knee Score; SD, standard deviation; SMD, standardized mean difference; WOMAC, Western Ontario and McMaster Universities Osteoarthritis Index.

**Supplementary Table S3. Fixed 48-month medial JSN endpoint audit.**

***Panel A. Baseline-to-48-month medial JSN grade transitions in the final analytic cohort***

| Baseline medial JSN grade | 48-month medial JSN grade | Knees | Participants represented | Full-grade progression events |
| --- | --- | --- | --- | --- |
| 0.0 | 0.0 | 1,958 | 1,675 | 0 |
| 0.0 | 1.0 | 71 | 68 | 71 |
| 0.0 | 1.2 | 4 | 4 | 4 |
| 0.0 | 1.4 | 1 | 1 | 1 |
| 0.0 | 2.0 | 45 | 42 | 45 |
| 0.0 | 2.2 | 6 | 6 | 6 |
| 0.0 | 2.4 | 1 | 1 | 1 |
| 0.0 | 2.6 | 3 | 3 | 3 |
| 0.0 | 3.0 | 4 | 4 | 4 |
| 1.0 | 0.0 | 1 | 1 | 0 |
| 1.0 | 1.0 | 433 | 389 | 0 |
| 1.0 | 1.2 | 18 | 17 | 0 |
| 1.0 | 1.4 | 7 | 7 | 0 |
| 1.0 | 1.6 | 1 | 1 | 0 |
| 1.0 | 2.0 | 66 | 64 | 66 |
| 1.0 | 2.2 | 9 | 9 | 9 |
| 1.0 | 2.4 | 9 | 9 | 9 |
| 1.0 | 2.6 | 5 | 5 | 5 |
| 1.0 | 2.8 | 1 | 1 | 1 |
| 1.0 | 3.0 | 6 | 6 | 6 |
| 2.0 | 2.0 | 112 | 110 | 0 |
| 2.0 | 2.2 | 22 | 22 | 0 |
| 2.0 | 2.4 | 27 | 27 | 0 |
| 2.0 | 2.6 | 19 | 19 | 0 |
| 2.0 | 2.8 | 11 | 11 | 0 |
| 2.0 | 3.0 | 59 | 58 | 59 |

***Panel B. Distribution of baseline-to-48-month medial JSN change***

| Medial JSN change | Knees | Primary full-grade event | Partial-or-full sensitivity event |
| --- | --- | --- | --- |
| −1.0 | 1 | No | No |
| 0.0 | 2,503 | No | No |
| 0.2 | 40 | No | Yes |
| 0.4 | 34 | No | Yes |
| 0.6 | 20 | No | Yes |
| 0.8 | 11 | No | Yes |
| 1.0 | 196 | Yes | Yes |
| 1.2 | 13 | Yes | Yes |
| 1.4 | 10 | Yes | Yes |
| 1.6 | 5 | Yes | Yes |
| 1.8 | 1 | Yes | Yes |
| 2.0 | 51 | Yes | Yes |
| 2.2 | 6 | Yes | Yes |
| 2.4 | 1 | Yes | Yes |
| 2.6 | 3 | Yes | Yes |
| 3.0 | 4 | Yes | Yes |
| Total | **2,899** | **290 events (10.0%)** | **395 events (13.6%)** |

***Note:*** The endpoint was reconstructed directly from paired baseline and fixed 48-month central medial JSN grades within the same longitudinal reading project. Primary progression required an increase of at least 1.0 grade. The partial-or-full sensitivity endpoint included any positive change. Decimal values represent within-grade readings in the source central assessments. Participant counts in Panel A are not additive across transitions because bilateral knees from the same participant can occupy different rows. JSN, joint-space narrowing.

**Supplementary Table S4. Complete inventory of the 115 individual baseline MOAKS predictors used in RMO and SMR.**

| Feature | Official descriptive category | Nonmissing knees, final cohort | Nonmissing knees, training | Unique observed values, training | Used as raw model predictor |
| --- | --- | --- | --- | --- | --- |
| V00MCMFLA | Cartilage morphology | 2898 | 2050 | 9 | Yes |
| V00MCMFLC | Cartilage morphology | 2899 | 2050 | 8 | Yes |
| V00MCMFLP | Cartilage morphology | 2899 | 2050 | 8 | Yes |
| V00MCMFMA | Cartilage morphology | 2898 | 2050 | 9 | Yes |
| V00MCMFMC | Cartilage morphology | 2899 | 2050 | 10 | Yes |
| V00MCMFMP | Cartilage morphology | 2899 | 2050 | 6 | Yes |
| V00MCMPL | Cartilage morphology | 2896 | 2048 | 10 | Yes |
| V00MCMPM | Cartilage morphology | 2897 | 2049 | 9 | Yes |
| V00MCMTLA | Cartilage morphology | 2898 | 2050 | 4 | Yes |
| V00MCMTLC | Cartilage morphology | 2898 | 2050 | 8 | Yes |
| V00MCMTLP | Cartilage morphology | 2898 | 2050 | 9 | Yes |
| V00MCMTMA | Cartilage morphology | 2899 | 2050 | 7 | Yes |
| V00MCMTMC | Cartilage morphology | 2899 | 2050 | 8 | Yes |
| V00MCMTMP | Cartilage morphology | 2899 | 2050 | 7 | Yes |
| V00MACLBML | Cruciate-ligament/patellar-tendon findings | 2460 | 1749 | 2 | Yes |
| V00MACLRP | Cruciate-ligament/patellar-tendon findings | 2460 | 1749 | 2 | Yes |
| V00MACLTR | Cruciate-ligament/patellar-tendon findings | 2460 | 1749 | 4 | Yes |
| V00MPCLBML | Cruciate-ligament/patellar-tendon findings | 1795 | 1275 | 2 | Yes |
| V00MPCLRP | Cruciate-ligament/patellar-tendon findings | 1795 | 1275 | 1 | Yes |
| V00MPCLTR | Cruciate-ligament/patellar-tendon findings | 1795 | 1275 | 2 | Yes |
| V00MPTSIG | Cruciate-ligament/patellar-tendon findings | 1857 | 1313 | 2 | Yes |
| V00MANSBUR | Ganglion/popliteal cysts and bursitis | 1860 | 1314 | 2 | Yes |
| V00MGCACL | Ganglion/popliteal cysts and bursitis | 1860 | 1314 | 2 | Yes |
| V00MGCOTH | Ganglion/popliteal cysts and bursitis | 1860 | 1314 | 2 | Yes |
| V00MGCPCL | Ganglion/popliteal cysts and bursitis | 1860 | 1314 | 2 | Yes |
| V00MGCSM | Ganglion/popliteal cysts and bursitis | 1860 | 1314 | 2 | Yes |
| V00MGCTIB | Ganglion/popliteal cysts and bursitis | 1860 | 1314 | 2 | Yes |
| V00MGSST | Ganglion/popliteal cysts and bursitis | 1860 | 1314 | 1 | Yes |
| V00MIPBUR | Ganglion/popliteal cysts and bursitis | 1860 | 1314 | 2 | Yes |
| V00MPOPCYS | Ganglion/popliteal cysts and bursitis | 1860 | 1314 | 2 | Yes |
| V00MPPBUR | Ganglion/popliteal cysts and bursitis | 1860 | 1314 | 2 | Yes |
| V00MITBSIG | Iliotibial-band signal | 561 | 406 | 1 | Yes |
| V00MMHLA | Meniscal findings | 2460 | 1749 | 1 | Yes |
| V00MMHLB | Meniscal findings | 2460 | 1749 | 2 | Yes |
| V00MMHLP | Meniscal findings | 2460 | 1749 | 1 | Yes |
| V00MMHMA | Meniscal findings | 2460 | 1749 | 1 | Yes |
| V00MMHMB | Meniscal findings | 2460 | 1749 | 2 | Yes |
| V00MMHMP | Meniscal findings | 2460 | 1749 | 2 | Yes |
| V00MMRTL | Meniscal findings | 2899 | 2050 | 2 | Yes |
| V00MMRTM | Meniscal findings | 2899 | 2050 | 2 | Yes |
| V00MMSLA | Meniscal findings | 2460 | 1749 | 2 | Yes |
| V00MMSLB | Meniscal findings | 2460 | 1749 | 2 | Yes |
| V00MMSLP | Meniscal findings | 2460 | 1749 | 2 | Yes |
| V00MMSMA | Meniscal findings | 2460 | 1749 | 2 | Yes |
| V00MMSMB | Meniscal findings | 2460 | 1749 | 2 | Yes |
| V00MMSMP | Meniscal findings | 2460 | 1749 | 2 | Yes |
| V00MMTLA | Meniscal findings | 2897 | 2050 | 7 | Yes |
| V00MMTLB | Meniscal findings | 2899 | 2050 | 8 | Yes |
| V00MMTLP | Meniscal findings | 2897 | 2050 | 8 | Yes |
| V00MMTMA | Meniscal findings | 2897 | 2050 | 5 | Yes |
| V00MMTMB | Meniscal findings | 2899 | 2050 | 8 | Yes |
| V00MMTMP | Meniscal findings | 2897 | 2050 | 8 | Yes |
| V00MMXLA | Meniscal findings | 2458 | 1747 | 3 | Yes |
| V00MMXLL | Meniscal findings | 2898 | 2049 | 4 | Yes |
| V00MMXMA | Meniscal findings | 2460 | 1749 | 4 | Yes |
| V00MMXMM | Meniscal findings | 2897 | 2048 | 4 | Yes |
| V00MOSFLA | Osteophytes | 1943 | 1375 | 4 | Yes |
| V00MOSFLC | Osteophytes | 1943 | 1375 | 4 | Yes |
| V00MOSFLP | Osteophytes | 1943 | 1375 | 4 | Yes |
| V00MOSFMA | Osteophytes | 1942 | 1375 | 4 | Yes |
| V00MOSFMC | Osteophytes | 1943 | 1375 | 4 | Yes |
| V00MOSFMP | Osteophytes | 1943 | 1375 | 4 | Yes |
| V00MOSPI | Osteophytes | 1943 | 1375 | 4 | Yes |
| V00MOSPL | Osteophytes | 1943 | 1375 | 4 | Yes |
| V00MOSPM | Osteophytes | 1943 | 1375 | 4 | Yes |
| V00MOSPS | Osteophytes | 1942 | 1375 | 4 | Yes |
| V00MOSTL | Osteophytes | 1943 | 1375 | 4 | Yes |
| V00MOSTM | Osteophytes | 1943 | 1375 | 4 | Yes |
| V00MBMNFLA | Subregional bone marrow lesions | 2460 | 1749 | 5 | Yes |
| V00MBMNFLC | Subregional bone marrow lesions | 2460 | 1749 | 3 | Yes |
| V00MBMNFLP | Subregional bone marrow lesions | 2460 | 1749 | 3 | Yes |
| V00MBMNFMA | Subregional bone marrow lesions | 2459 | 1749 | 4 | Yes |
| V00MBMNFMC | Subregional bone marrow lesions | 2460 | 1749 | 4 | Yes |
| V00MBMNFMP | Subregional bone marrow lesions | 2460 | 1749 | 4 | Yes |
| V00MBMNPL | Subregional bone marrow lesions | 2460 | 1749 | 5 | Yes |
| V00MBMNPM | Subregional bone marrow lesions | 2459 | 1749 | 5 | Yes |
| V00MBMNSS | Subregional bone marrow lesions | 2460 | 1749 | 5 | Yes |
| V00MBMNTLA | Subregional bone marrow lesions | 2460 | 1749 | 2 | Yes |
| V00MBMNTLC | Subregional bone marrow lesions | 2460 | 1749 | 3 | Yes |
| V00MBMNTLP | Subregional bone marrow lesions | 2460 | 1749 | 2 | Yes |
| V00MBMNTMA | Subregional bone marrow lesions | 2460 | 1749 | 3 | Yes |
| V00MBMNTMC | Subregional bone marrow lesions | 2460 | 1749 | 4 | Yes |
| V00MBMNTMP | Subregional bone marrow lesions | 2460 | 1749 | 3 | Yes |
| V00MBMPFLA | Subregional bone marrow lesions | 2460 | 1749 | 4 | Yes |
| V00MBMPFLC | Subregional bone marrow lesions | 2459 | 1748 | 4 | Yes |
| V00MBMPFLP | Subregional bone marrow lesions | 2459 | 1748 | 4 | Yes |
| V00MBMPFMA | Subregional bone marrow lesions | 2460 | 1749 | 4 | Yes |
| V00MBMPFMC | Subregional bone marrow lesions | 2460 | 1749 | 4 | Yes |
| V00MBMPFMP | Subregional bone marrow lesions | 2460 | 1749 | 4 | Yes |
| V00MBMPPL | Subregional bone marrow lesions | 2459 | 1748 | 4 | Yes |
| V00MBMPPM | Subregional bone marrow lesions | 2459 | 1748 | 4 | Yes |
| V00MBMPSS | Subregional bone marrow lesions | 2459 | 1748 | 5 | Yes |
| V00MBMPTLA | Subregional bone marrow lesions | 2460 | 1749 | 2 | Yes |
| V00MBMPTLC | Subregional bone marrow lesions | 2460 | 1749 | 4 | Yes |
| V00MBMPTLP | Subregional bone marrow lesions | 2460 | 1749 | 4 | Yes |
| V00MBMPTMA | Subregional bone marrow lesions | 2460 | 1749 | 4 | Yes |
| V00MBMPTMC | Subregional bone marrow lesions | 2460 | 1749 | 4 | Yes |
| V00MBMPTMP | Subregional bone marrow lesions | 2460 | 1749 | 4 | Yes |
| V00MBMSFLA | Subregional bone marrow lesions | 2898 | 2050 | 4 | Yes |
| V00MBMSFLC | Subregional bone marrow lesions | 2897 | 2049 | 4 | Yes |
| V00MBMSFLP | Subregional bone marrow lesions | 2897 | 2049 | 4 | Yes |
| V00MBMSFMA | Subregional bone marrow lesions | 2898 | 2050 | 4 | Yes |
| V00MBMSFMC | Subregional bone marrow lesions | 2898 | 2050 | 4 | Yes |
| V00MBMSFMP | Subregional bone marrow lesions | 2898 | 2050 | 4 | Yes |
| V00MBMSPL | Subregional bone marrow lesions | 2897 | 2049 | 4 | Yes |
| V00MBMSPM | Subregional bone marrow lesions | 2897 | 2049 | 4 | Yes |
| V00MBMSSS | Subregional bone marrow lesions | 2896 | 2048 | 4 | Yes |
| V00MBMSTLA | Subregional bone marrow lesions | 2898 | 2050 | 3 | Yes |
| V00MBMSTLC | Subregional bone marrow lesions | 2898 | 2050 | 4 | Yes |
| V00MBMSTLP | Subregional bone marrow lesions | 2898 | 2050 | 4 | Yes |
| V00MBMSTMA | Subregional bone marrow lesions | 2898 | 2050 | 4 | Yes |
| V00MBMSTMC | Subregional bone marrow lesions | 2898 | 2050 | 4 | Yes |
| V00MBMSTMP | Subregional bone marrow lesions | 2898 | 2050 | 4 | Yes |
| V00MEFFWK | Synovitis/effusion | 2460 | 1749 | 4 | Yes |
| V00MSYIC | Synovitis/effusion | 2460 | 1749 | 4 | Yes |

***Note:*** All 115 listed variables entered the raw predictor matrix. Missing values were imputed using training-set estimates, and preprocessing parameters were fitted using the training partition only. Domain-level MOAKS sums shown in main Table 1 were used only for descriptive presentation and did not replace these individual predictors in model fitting. MOAKS, MRI Osteoarthritis Knee Score; RMO, raw MOAKS model; SMR, structured MRI/radiographic model.

**Supplementary Table S5. Secondary comparators, neural-network ablations, predictor-coding sensitivity, and endpoint sensitivity.**

***Panel A. Enhanced clinical-radiographic comparator***

| Model | Inputs | Test AUPRC | Test AUROC | Test Brier score |
| --- | --- | --- | --- | --- |
| Enhanced clinical-radiographic comparator | Age, sex, BMI, baseline WOMAC pain, KL grade, medial JSN, and lateral JSN | 0.251 | 0.759 | 0.205 |
| Enhanced comparator plus MOAKS | Enhanced comparator inputs plus the individual baseline MOAKS predictors | 0.355 | 0.813 | 0.184 |

**Paired difference, enhanced comparator plus MOAKS minus enhanced comparator:** AUPRC +0.104 (95% patient-cluster CI +0.026 to +0.215), AUROC +0.053 (+0.002 to +0.105), and Brier score −0.021 (−0.030 to −0.012).

***Panel B. Numeric-ordinal primary models and categorical-coding sensitivity***

| Model | Predictor coding | Test AUPRC | Test AUROC | Test Brier score |
| --- | --- | --- | --- | --- |
| RMO | Numeric ordinal, primary | 0.412 | 0.805 | 0.188 |
| RMO | One-hot categorical, sensitivity | 0.368 | 0.815 | 0.164 |
| SMR | Numeric ordinal, primary | 0.345 | 0.801 | 0.196 |
| SMR | One-hot categorical, sensitivity | 0.417 | 0.837 | 0.170 |

***Panel C. Neural-network ablations***

| Model | Inputs | Test AUPRC | Test AUROC | Test Brier score |
| --- | --- | --- | --- | --- |
| Tabular multilayer perceptron | Same MOAKS and radiographic predictors as SMR | 0.305 | 0.753 | 0.167 |
| X-ray-only ResNet50 | Baseline radiograph only | 0.209 | 0.696 | 0.226 |
| XMF | Baseline radiograph plus SMR structured predictors | 0.301 | 0.768 | 0.185 |

***Panel D. Endpoint sensitivity***

| Endpoint | Events, n (%) | RBM test AUPRC | RMO test AUPRC | SMR test AUPRC |
| --- | --- | --- | --- | --- |
| Full-grade worsening, primary | 290 (10.0%) | 0.220 | 0.412 | 0.345 |
| Any partial-or-full worsening, sensitivity | 395 (13.6%) | 0.347 | 0.579 | 0.530 |

***Note:*** All models retained the fixed participant assignments. Hyperparameters were selected using validation AUPRC. The categorical analysis changed predictor representation but not the endpoint or participant partitions. The partial-or-full endpoint sensitivity retained the same structured predictors and participant assignments and refitted RBM, RMO, and SMR. The neural-network ablations are architecture-specific and do not test end-to-end learning from source MRI images. AUPRC, area under the precision–recall curve; AUROC, area under the receiver-operating-characteristic curve; BMI, body mass index; CI, confidence interval; JSN, joint-space narrowing; KL, Kellgren–Lawrence; MOAKS, MRI Osteoarthritis Knee Score; RBM, radiographic baseline model; RMO, raw MOAKS model; SMR, structured MRI/radiographic model; WOMAC, Western Ontario and McMaster Universities Osteoarthritis Index; XMF, X-ray plus MOAKS fusion model.
